# The First Model-Based Geostatistical Map of Anaemia

**DOI:** 10.1371/journal.pmed.1001039

**Published:** 2011-06-07

**Authors:** Abdisalan M. Noor

**Affiliations:** Malaria Public Health and Epidemiology Group, Centre for Geographic Medicine–Coast, Kenya Medical Research Institute–University of Oxford-Wellcome Trust Research Programme, Nairobi, Kenya

## Abstract

Abdisalan Noor discusses new research in <i>PLoS Medicine<I> that used model-based geostatistics to investigate the risks of anemia among preschool-aged children in West Africa that were attributable to malnutrition, malaria, and helminth infections.

**Linked Research Article**

This Perspective discusses the following new study published in *PLoS Medicine*:

Soares Magalhães RJ, Clements ACA (2011) Mapping the Risk of Anaemia in Preschool-Age Children: The Contribution of Malnutrition, Malaria, and Helminth Infections in West Africa. PLoS Med 8: e1000438. doi:10.1371/journal.pmed.1000438

Ricardo Soares Magalhães and colleagues used national cross-sectional household-based demographic health surveys to map the distribution of anemia risk in preschool children in Burkina Faso, Ghana, and Mali.

Anaemia is the most common blood disorder globally, is a major underlying cause of death among children and pregnant women in the developing world, and because it is most prevalent in the poorest communities, represents a useful marker of social and economic marginalization [1]–[3]. The aetiology of anaemia is often multi-factorial including nutritional deficiencies, parasitic and inflammatory diseases, haemorrhage, and genetic defects in the molecular structure of the haemoglobin (Hb) [3]. Many of these risk factors for anaemia co-exist in communities and affect individuals in composite ways not adequately understood. In Africa three dominant contributors to anaemia in young children are malaria, helminth infections, and iron-deficient diets. To date, only national-level estimates of anaemia burden developed by the World Health Organization are available to guide control [3]. There are limited data on within-country variations and no detailed information on the uncertainty around these estimates or on the combination of causal factors important to a country.

The article published in *PLoS Medicine* this week by Magalhães and Clements [4] explores the use of model-based geostatistics to investigate the risks of anaemia and mean Hb concentrations among preschool-age children (aged 1–4 years) attributable to malnutrition, malaria, and helminth infections in three West African countries (Burkina Faso, Ghana, and Mali). The authors develop high resolution maps of the geographic distribution of anaemia accounting for these factors and compute the estimated number of anaemic children. The advantage of using model-based geostatistics to model anaemia is that it allows the use of the survey data from a sample of locations to predict continuous surfaces of risk, informed by environmental and demographic covariates, but in a way that takes into account the sample size and spatial characteristic of the data to robustly assess uncertainty in the modelled outputs. The study identified malnutrition as the main driver of anaemia, followed by malaria and helminth infections, accounting for a population attributable fraction (PAF) of almost 60% of all anaemia. The mean Hb concentration increased with age and varied by country. The continuous surfaces of anaemia prevalence and Hb concentration show interesting variations within the national borders that allow for the computation of the location and radius of foci of high risks. The estimated case burden for 2011 was similar to that provided by the World Health Organization in its 1993–2005 worldwide anaemia prevalence report [3].

## The Public Health Relevance and Limitations

The application of model-based geostatistics to national sample survey data on anaemia is an important advance in our understanding of the geography of risk and will provide a more robust framework for computing disease burdens and attributable disability adjusted life years (DALYs). However, for ministries of health, which require disease mapping to guide control and prevention, it is less clear how these new maps will provide enough information on what must be done to reduce the anaemia burdens. Typically, these maps will tell us where the risks are high, moderate, or low, but without knowing the underlying multi-factorial—and most certainly interacting—causes of anaemia, it becomes difficult to determine which combination of interventions is most effective. The authors attempt to partly address this challenge by computing the anaemia PAF of malnutrition, malaria, and helminth infections. As recognised by the authors, PAF can be highly biased depending on whether important confounders are missing from the analysis. HIV infection, haemoglobinopathies, and several other contributors were not included in this study in estimating PAF. It is likely that the inclusion of these contributory factors would decrease the PAF of malnutrition, malaria, and helminth infections and therefore the cost-effectiveness of interventions to control these causes based on the current 60% PAF estimated in this study.

The second limitation is one that is specific to the currency of the data used. In the past decade, specifically within the last six years, there has been substantial progress in governance, economics, and funding for control of tropical infections, as well as increased iron and micronutrient supplementation, which would have impacted on these anaemia risk factors in the study countries [3],[5]–[8]. The data used for this study are relatively old and represent a time when investments in malnutrition, malaria, and helminth control were minimal, the coverage of insecticide-treated nets (providing protection against malaria-carrying mosquitoes) was low, and a failing drug, sulphadoxine pyremethamine, was the first line treatment for uncomplicated malaria [6],[8],[9]. By 2008, Ghana has halved it incidence of poverty and increased its gross domestic product by almost 60% relative to 2002, and chronic malnutrition has dropped considerably [10]. Investment in malaria control increased from US$900,000 in 2003 to US$54 million by 2009, while school feeding and deworming programmes and community nutrition programmes have been scaled up [5],[7],[8]. Coverage of insecticide-treated nets among children under the age of five years was 30% in 2008 compared to only 4% in 2003, and artemisinin combination therapies replaced sulphadoxine pyremethamine as the recommended drug for uncomplicated malaria treatment [7]. Less impressive but equally significant is the progress made in the two Sahelian countries of Burkina Faso and Mali in economic growth and the control of malnutrition, malaria, and helminth infections [5],[7],[8], although a series of recent famines have contributed to chronic food shortages in large areas. In Mali, the government has established large agricultural schemes such as the Rice Initiative to alleviate food insecurity [11]. These changes are likely to have affected the anaemia case burden, its geographic distribution, and the fractions attributable to the three main causal factors.

## Future Work

More contemporary survey data, updated maps of malaria and helminth infections, and information on other important causal factors are therefore required to improve the value of the anaemia maps for efficient control planning. Data from national HIV/AIDS indicator surveys, recent work on mapping the distribution of sickle cell disease [12], and ongoing work on the cartography of other blood disorders (Malaria Atlas Project; http://www.map.ox.ac.uk) could potentially address this challenge. The use of the malaria, helminth, and population distribution maps to define the burden of anaemia is novel, but these products are associated with uncertainties, and as yet there are no clear approaches to understanding their combined effect on the primary measure and whether they are additive or multiplicative. Future work must address these data and methodological challenges.

## Abbreviations

Hb: haemoglobin
PAF: population attributable fraction
